# Handgrip strength assessment in breast cancer patients: validity and reliability of the Activ5 dynamometer

**DOI:** 10.1007/s00520-025-09895-8

**Published:** 2025-09-20

**Authors:** José Pino-Ortega, Rafael Carvajal-Espinoza, Boryi Becerra-Patiño, Aaron Gómez-Parra, Adrián Moreno-Villanueva

**Affiliations:** 1 BIOVETMED & SPORTSCI Research Group, Faculty of Sports Sciences, University of Murcia, San Javier, Spain; 2 School of Physical Education and Sports, University of Costa Rica, San José, 11501-2060 Costa Rica; 3 Physical Activity and Sport Management and Pedagogy Group (GPAFD), Faculty of Physical Education, National Pedagogical University, Bogotá, Colombia; 4 Faculty of Sport Science, University of Murcia, Murcia, Spain; 5 Department of Health Sciences, University Isabel I of Castille, Burgos, Spain

**Keywords:** Strength, Dynamometer, Validity, Reliability, Breast cancer

## Abstract

Hand dynamometry is a widely accepted method for measuring muscle strength and serves as a crucial clinical indicator, particularly in populations with chronic health conditions, such as cancer. Despite its growing use, no prior study has evaluated the validity of the Activ5 portable dynamometer in individuals with cancer. This study aimed to determine the reliability and validity of the Activ5 for assessing handgrip strength (HGS) in a population of female cancer survivors. The sample consisted of 22 women (mean age 53.91 ± 5.93 years; body mass 65.85 ± 14.52 kg; height 1.65 ± 0.04 m), who were assessed across two testing sessions in a cross-sectional concordance design. Reliability and validity were analyzed using Pearson correlation coefficients, intraclass correlation coefficients (ICC), and Lin’s concordance coefficient. Results demonstrated very strong correlations between the Activ5 and the gold-standard Jamar dynamometer. For the right hand, *r* = 0.895 (*p* = 0.001), and for the left hand, *r* = 0.893 (*p* = 0.001). Lin’s ICC for the right hand was 0.995 (95% CI: 0.974–1.016), and for the left hand 0.990 (95% CI: 0.959–1.020). These findings indicate excellent agreement and support the use of the Activ5 as a valid and reliable tool for evaluating HGS in cancer survivors. Its portability and ease of use make it a practical option for both clinical and research settings.

## Introduction

In medicine, one of the significant current clinical challenges is muscle dysfunction associated with cancer, even in patients with low tumor burden [12, 49]. Multiple conditions can contribute to impaired muscle function, including aging, comorbidities, malnutrition, inadequate dietary intake, physical inactivity, sedentary behavior, and the adverse effects of oncological treatments [1, 17, 29].

Observational studies have suggested that physical activity (PA) can reduce cancer-specific mortality, particularly in breast cancer (BC) patients [44], while others have explored the impact of sarcopenia on mortality risk in BC survivors [42, 48]. Advances in early detection and treatment have improved survival rates,however, adverse effects on nutritional status, inflammation, and body composition remain frequent [4, 47]. These factors often lead to muscle mass loss and reduced strength, which compromise quality of life. As such, improving functional status and health-related quality of life is now a healthcare priority for breast cancer survivors [27].

Despite improvements in treatment, BC survivors often receive less follow-up and rehabilitation support compared to the general population [45]. Treatments such as surgery, chemotherapy, radiotherapy, hormonal therapy, and targeted therapy have been associated with decreased muscle strength, fatigue, and sarcopenia [20, 26]. Muscle strength, therefore, is an essential clinical parameter in the assessment and rehabilitation of cancer patients [10, 18, 46].

Hand grip strength (HGS), in particular, has emerged as a reliable, non-invasive, and cost-effective indicator of overall muscle strength [15]. In the general population, low HGS correlates with higher mortality rates, especially from cardiovascular, respiratory, and cancer-related causes [11, 12]. Among patients with advanced cancer, HGS is also associated with malnutrition, cognitive dysfunction, and poorer surgical outcomes [32, 39]. Prospective evidence also confirms that lower HGS is associated with all-cause mortality and reduced physical fitness in advanced cancer [21, 25].

Construct and criterion validity are population-dependent properties. In the case of cancer survivors (particularly breast cancer patients), there are well-documented alterations in muscle function, tone, fatigue levels, joint range of motion, and neuromuscular activation, all of which can influence the biomechanics and physiology of grip strength [9, 20, 26]. Therefore, a device validated in a healthy population must be re-evaluated in populations with distinct physiological and functional profiles to ensure that its measurements remain accurate, reliable, and clinically useful.

Given its clinical utility, efforts to improve the accessibility and implementation of HGS measurements are warranted. While the Jamar dynamometer is considered the gold standard, it is not always practical in all settings. The Activ5 device is a portable, low-cost alternative that provides real-time strength measurement and individualized feedback. However, no studies have yet assessed its reliability and validity in cancer populations. Therefore, this study aimed to evaluate the reliability and validity of the Activ5 dynamometer for assessing grip strength in female cancer survivors.

## Method

### Study design

The present research evaluated the reliability and concurrent validity of the portable Activ5® dynamometer (Activbody Inc., San Diego, California, USA) by comparing it to the gold-standard hydraulic hand dynamometer, the Jamar. The guidelines for reporting reliability and agreement studies (GRRAS) [28] were followed. The project was approved by the University of Murcia ethics committee (ID: 3495/2021).

### Instruments

**Fig. 1:**
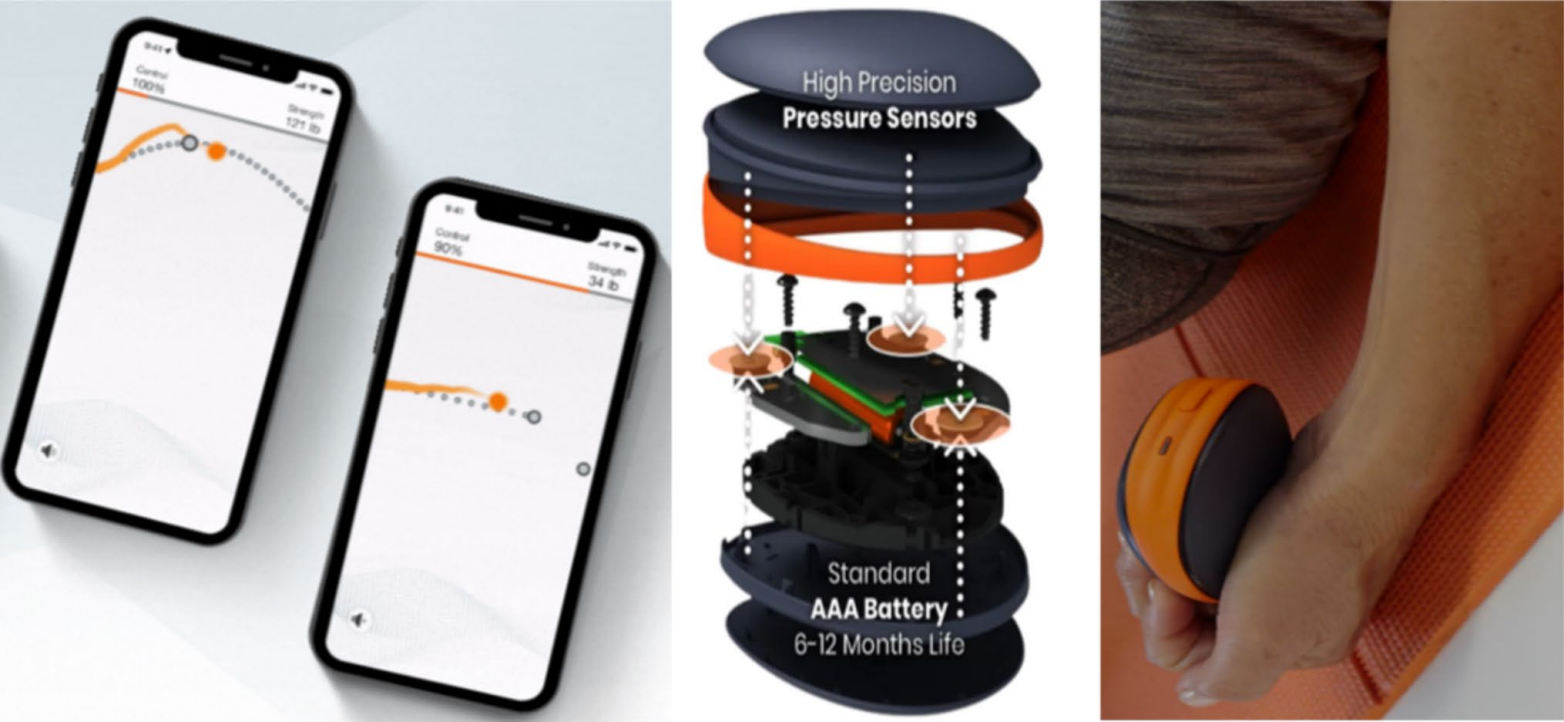
Activ5 force dynamometer. Source: https://activ5.com/

The instrument used to evaluate HGS was the Activ5 dynamometer (Paterson Medical, Green Bay, WI, USA), which features a digital grip and measures the grip strength generated by the individual, projecting the results to a mobile application (Activ5) via Bluetooth technology, as shown in Fig. 1. The instrument’s operating system is compatible with iOS and Android technology. The main characteristics of the instrument (length x width x height) [mm] are 95 × 78x33, and it has a sampling frequency of 10 Hz, a maximum design/production load at full scale (FS) of 90 kg, and an accuracy of ± (0.635 kg + 5% of the applied force) [8, 33]. On the other hand, the Jamar dynamometer is a hand-held device that can be easily calibrated with a specific weight [30]. This dynamometer has been utilized in various studies with available normative data [6, 7, 43]. The comparison was made with the hand-held dynamometer (Jamar Hand Dynamometer, IL, USA).

### Participants

The sample consisted of women with breast cancer (*n* = 22) who voluntarily agreed to participate in the study, as shown in Table 1. Regarding clinical status, 45% of participants (*n* = 10) were in a disease-free interval, 35% (*n* = 8) were undergoing adjuvant treatment, and 5% (*n* = 1) each were classified as non-metastatic disease-free, recent immunological treatment, advanced disease, and unspecified clinical status. In terms of lymphedema, 91% (*n* = 20) showed no signs, while 9% (*n* = 2) presented with lymphedema. Concerning the time since treatment initiation, 41% (*n* = 9) had less than 6 months, 32% (*n* = 7) between 6 and 12 months, 18% (*n* = 4) between 1 and 2 years, 5% (*n* = 1) more than 3 years, and another 5% (*n* = 1) at least 5 years. Before beginning the evaluations, an informational session was held where the purposes and scope of the study, the tests to be conducted, the procedure, and the protocol were explained. All participants were familiar with the protocol prior to the evaluations being performed. The inclusion criteria for this study were: (1) signing the informed consent forms; (2) no significant cardiovascular disease or active infection at the time of the evaluation; (3) being in an advanced stage; (4) being over 18 years old; (5) surgery on either left or right breast. The exclusion criteria were: (1) presence or history of heart disease; (2) undergoing ongoing antibiotic therapy or showing clinical signs of acute infection; (3) having a second cancer diagnosis within the last 5 years of the study; (4) having current or recent hand injuries or having undergone hand surgeries in the past 6 months; (5) experiencing discomfort during the tests. Ethical approval was obtained from the Ethics Committee of UMU (ID: M10/2023/004). All procedures were established in accordance with the guidelines of the Declaration of Helsinki, which involves human participants [49].

**Table 1:** Sample characteristics (x̄ ± SD)

n	Age (y)	Weight (kg)	Height (m)	BMI (kg/m^2^)
22	53.91 ± 5.93	65.85 ± 14.52	1.65 ± 0.04	23.9 ± 4.25
Los valores se expresan como media ± SD				

*SD* standard deviation, *y* years, *kg* kilograms, *m* centimeters, *BMI* Body mass index

### Procedure

The measurements were taken by a researcher experienced in using the device at the University of Murcia facilities. The aim was to ensure that the measurements were always taken under the same conditions: (morning time), and ambient temperature (22–27 °C). These evaluations were conducted at two separate times, with a 5-day interval between them. Several factors were considered in carrying out the evaluations: (1) materials used; (2) adequacy of the space; (3) organization of participants for evaluation; (4) evaluation protocol; (5) time allocated for administering the evaluations; (6) methods for processing information.

The procedure used to evaluate HGS was as described by Magni et al. [30]. For this, the evaluated person is seated with their feet resting on the floor, their arm unsupported, their shoulder in adduction, their elbow flexed at 90°, and their wrist and forearm in a neutral position. The wrist should be supported between 0 and 30° of dorsiflexion and 0 to 10° of ulnar deviation. In addition, a protocol of three repetitions of 5-s maximum contractions with 30-s intervals was followed for a correct evaluation, and an average of the three attempts was made to calculate the data. After completing the measurement with one hand, the evaluation proceeded with the other. The order of instrument application was randomized for each participant, with some starting the evaluation with the Activ5 dynamometer and others with the Jamar dynamometer. Moreover, the tests with both instruments were conducted on the same day with a 12-min interval, as described in another study [30]. All participants, without exception, were evaluated in a seated position to ensure the homogenization of results [3]. Thus, the Activ5 dynamometer was placed in the right hand and then in the left hand of the evaluated person while the evaluator held it without adjusting, as other studies have recommended [31, 40].

### Statistical analysis

A Pearson correlation analysis was performed to identify the correlation between the Jamar dynamometer and the Activ5 device. The Lin coefficient was also obtained for this purpose. Subsequently, the instrument’s reliability was assessed using the test–retest technique, performing the Intraclass Correlation Coefficient (ICC) analysis for the three measurements of each participant. The analyses were conducted using the SPSS statistical software version 25 (SPSS Inc., Chicago, IL, USA).

## Results

Participants and Grip Strength Measurement Reliability. The averages of the test results obtained for both devices are shown in Table 2.

**Table 2:** Mean and standard deviation of grip strength results for Jamar and Activ5 dynamometers (x̄ ± SD)

N	Jamar Dynamometer Right-Hand (Kg)	Activ5 Right hand (Kg)	Delta (Δ)	E.S	Jamar Dynamometer Left-hand (Kg)	Activ5 left hand (Kg)	Delta (Δ)	E.S
22	22.59 ± 3.65	20.03 ± 4.05	2.56 ± 1.80	0.6	21.91 ± 3.58	20.27 ± 3.95	1.63 ± 1.77	0.4

*x̄* Mean, *SD* standard deviation, *kg* kilograms, *N* sample, *E.S.* effect size

### Correlation analysis

**Fig. 2:**
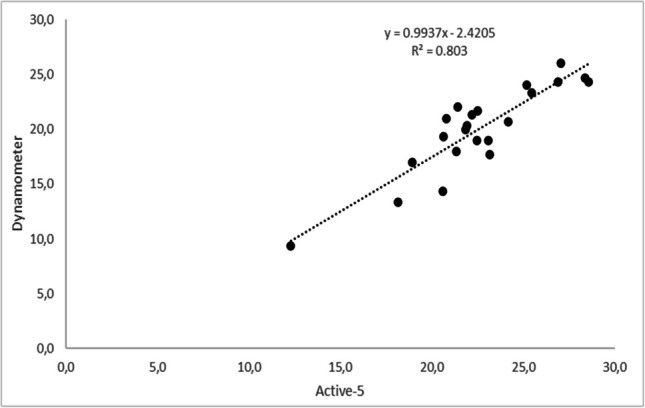
Correlation between Jamar Dynamometer and Activ-5 for the right hand

**Fig. 3:**
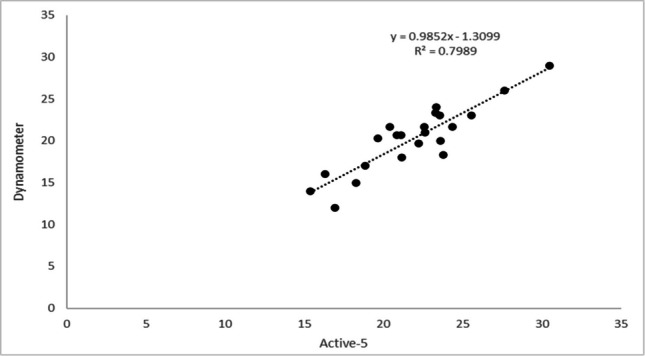
Correlation between Jamar Dynamometer and Activ5 for the left hand

The correlation analyses between the two devices showed strong positive relationships in the handgrip strength variable. In the right-hand condition, a value of *r* = 0.895 (*p* = 0.001) was obtained, and in the left-hand condition, a value of *r* = 0.893 (*p* = 0.001). Both correlations are statistically significant and reflect a robust relationship in which, as the values of one device increase, those of the other tend to do the same. These findings strengthen the consistency and relationship between the measurements of the devices (Figs. 2 and 3).

To assess the agreement between the Activ5 and Jamar dynamometers in measuring hand grip strength (HGS), Bland–Altman plots were constructed for both hands. This analysis allowed the evaluation of systematic bias and the limits of agreement between the two instruments.

For the right hand, the mean difference between devices was 2.56 kg, with 95% limits of agreement ranging from 1.08 kg to 8.12 kg. For the left hand, the mean difference was 1.63 kg, with 95% limits of agreement between − 0.57 kg and 6.37 kg. These values indicate a consistent positive bias, suggesting that the Activ5 tends to overestimate grip strength relative to the Jamar dynamometer.

**Fig. 4:**
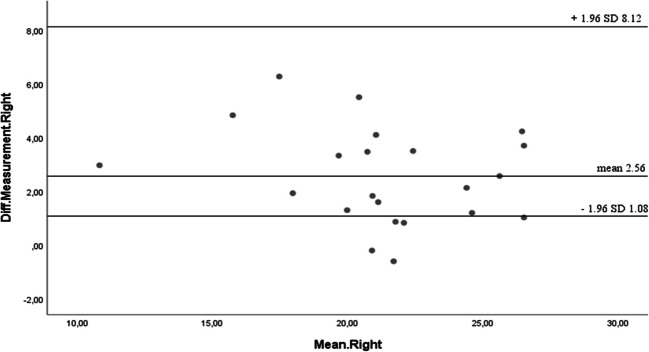
Bland–Altman plots the differences between the right hands (Units in Kg)

**Fig. 5:**
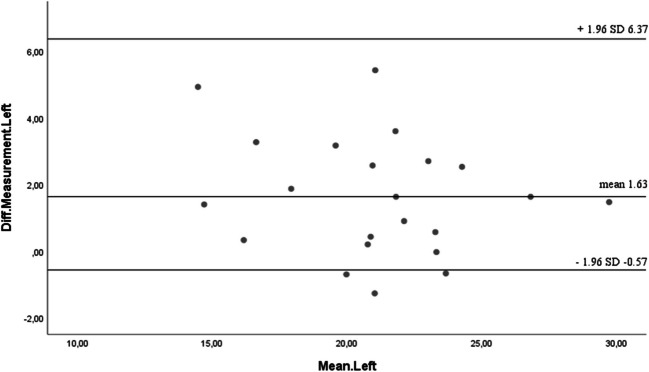
Bland–Altman plots the differences between the left hands (units in Kg)

Despite the observed bias, the majority of individual differences fell within the calculated limits of agreement, indicating an acceptable level of agreement for clinical and research use. The variability observed falls within a clinically relevant range, supporting the potential interchangeability of the two devices in this population while acknowledging the systematic difference between them (Figs. 4 and 5).

An analysis that complements the relationship between both measuring instruments is Lin’s Concordance Correlation Coefficient (CCC). This coefficient measures, in addition to reliability, the precision of the measurements. The calculation of this coefficient resulted in the right-hand condition: CCC = 0.995 with 95% confidence limits of 0.974–1.016 (lower–upper), while for the left-hand condition, it showed a CCC = 0.990 with 95% confidence limits of 0.959–1.020 (lower–upper). This result confirms the precision of the measurements from the instruments, assuming that both instruments measure hand grip strength in both conditions.

Reliability and consistency between measurements were evaluated using the Intraclass Correlation Coefficient (ICC) to analyze measurement variability. Three measurements were made for each subject, using both instruments and in both conditions (right hand and left hand). The results are presented in Table 3.

**Table 3:** Intra-class Correlation Coefficient (ICC) for Jamar Dynamometer and Activ5 under two conditions

	Jamar		Activ-5	
	Right	Left	Right	Left
ICC	0.933	0.953	0.973	0.966
Lower Limit	0.863	0.905	0.946	0.93
Upper Limit	0.97	0.979	0.988	0.985
Cronbach’s Alfa	0.933	0.953	0.973	0.966

*ICC* Intra-class correlation coefficient

In conducting this validation study, the guidelines for selecting health measurement instruments (COSMIN) [35] were considered, defining that the instrument measures the intended outcome [36].

## Discussion

The present study, which aimed to examine the validity and reliability of the Activ5 dynamometer in cancer patients, reveals that the Activ5 instrument demonstrates reliability when assessing grip strength in this population, yielding results similar to those obtained with the gold-standard dynamometer, the Jamar dynamometer. Thus, manual dynamometry showed good to excellent reliability for evaluating isometric muscle strength in the arm muscles of women with cancer.

The strong correlations observed between the Activ5 and Jamar devices (R^2^ = 0.80) indicate substantial agreement, with approximately 80% of the variance in measurements shared. The remaining unexplained variance may result from differences in device sensitivity, feedback modality, grip ergonomics, and calibration, as well as minor variations in participant effort or positioning. These discrepancies are expected when comparing digital and analog tools. Nonetheless, the strength of association supports the validity of the Activ5 as a practical alternative for assessing handgrip strength in clinical populations, such as cancer survivors.

The findings of this study reported high levels of reliability (ICC), as has been reported in other studies [6, 13, 14, 50]. The values reported by both instruments, Activ5 and Jamar, fell within confidence intervals of over 95%, demonstrating excellent test–retest reliability. To the best of our knowledge, this is the first study to evaluate the validity and reliability of the Activ5 instrument in patients with cancer.

The results indicate that as the evaluation of one instrument increased, so evaluated the other. Similar results have been reported in other studies when examining the validity of the Sport Experts® hand grip dynamometer, which measures grip strength continuity, and comparing it with the Takei and Baseline® dynamometers [19], as well as a study to determine the validity and reliability of two hand grip dynamometers, the Jamar instrument and the K-Force Grip [37].

The reduction of muscle strength (sarcopenia), loss of muscle mass (atrophy), and the decline in muscle strength associated with aging (sarcopenia) are factors that impact the prognosis of oncological processes [24, 34, 38]. Therefore, assessing HGS in the clinical setting is necessary, although limited by the high cost of the gold standard device (Jamar), which has led the scientific community to consider other alternatives with greater practicality and lower cost [40].

In this sense, the study conducted by Jiménez-Sánchez et al. [24] aimed to compare the precision, reliability, and concordance of two hand grip devices (Jamar Plus and Camry EH101) in colorectal cancer patients, finding that the precision and reliability of the measurements were high for both devices. However, the Bland–Altman analysis revealed a bias of 0.1 kg and limits of agreement from −5.3 to 5.4 kg in men and a bias of 1.5 kg and a limit of agreement from −2.2 to 5.3 kg in women. Meanwhile, in the present study, the right-hand measurement showed differences of 2.56 kg, with limits of agreement ranging from 1.08 to 8.12, and for the left hand, differences of 1.63 kg, with limits of agreement ranging from −0.57 to 6.37. This reveals that the limits of agreement fall within acceptable limits for both conditions, indicating a satisfactory concordance between each of the measurements. However, a positive systematic bias is observed in evaluating HGS for both the right and left hand, indicating that one of the instruments tends to overestimate grip strength. In this regard, the study by Savas et al. [43] in adults aged 60 and older concluded that the Jamar Plus instrument overestimates HGS in women by + 1.4 kg and nearly imperceptibly in men (−0.1 kg) when compared with the analog Jamar dynamometer, results that align with comparisons between two devices in the evaluation of HGS in cancer patients [24, 41]. Both the Activ5 dynamometer and the Jamar dynamometer in the present study demonstrated high reliability in the measurements taken with cancer patients, with both ICC and Cronbach’s alpha for the right hand = 0.973 and left hand = 0.966 for the Activ5, as well as for the Jamar dynamometer with right hand = 0.933 and left hand = 0.953.

For the present study, the maximum and mean force achieved by both dynamometers showed statistically significant correlations, demonstrating consistency in the established relationship for all measurements. The Jamar instrument (0.933 for the right hand and 0.953 for the left hand) showed lower ICC values than the Activ5 dynamometer (0.973 for the right hand and 0.966 for the left hand). These results do not align with those defined in the study by Jiménez-Sánchez et al. [24], where it was concluded that the Jamar Plus instrument showed slightly higher maximum and mean strength in cancer patients than the Camry dynamometer. These differences are also greater than those reported in other studies with adults over 50 years [22, 23].

However, it is important to clarify that the use of the Activ5 instrument in clinical evaluations for patients with different types of cancer should be done with caution, considering the estimate obtained in this study and referencing other studies that suggest the same when comparing HGS assessment between the K-Force and Jamar dynamometers [30].

In this regard, it is important to replicate this type of study in other population groups with diverse types of cancer, aiming to establish evaluation protocols that help understand the validity and reliability of the Activ5 instrument, especially since, being a portable instrument, its weight may cause different efforts or complications when operating the dynamometer, leading to additional stress on the joints when exerting force [2, 16]. For this reason, considering that both the Activ5 and Jamar dynamometers have different characteristics in terms of weight, structure, portability, ergonomics, and precision, further studies are needed to monitor HGS evaluation in various clinical settings, during recovery and in rehabilitation processes.

## Limitations

The study’s main limitations stem from the small sample size used to validate the instrument. In this regard, it is also necessary to consider that the Activ5 dynamometer was validated in a specific context, especially for women with breast cancer. The findings of this study cannot be extrapolated to patients with different types of cancer. Therefore, further studies are needed to confirm its application in clinical and research settings for patients with other types of cancer. For this reason, the results of this study should be interpreted with caution, as other studies have confirmed that the reliability of manual dynamometry varies in healthy population groups compared to clinical samples [5].

Future Perspectives and Practical Applications Although the sample evaluated in this study was women with breast cancer, data for such population groups are needed. The need to validate an instrument that can be portable and help resolve the evaluation of handgrip strength in the cancer population is justified. Such wireless connection instruments, which are easy to carry, could become daily-use tools for populations with low physical activity levels or those in a clinical setting [40], although there is a lower accuracy of this device above 40 kg. The validation of the instrument enables various research scenarios through longitudinal designs, acknowledging the utility of the Activ5 instrument.

## Conclusions

According to the results of this study, the digital Activ5 dynamometer demonstrated excellent intra- and inter-rater reliability for assessing handgrip strength (HGS) in cancer patients. Its use is considered for its practicality, portability, low cost, and precision in measuring hand grip strength in real time. This justifies the validation and reliability research of new instruments that emerge on the market to measure grip strength in real-time, explaining the importance of establishing their use in different population samples.

## Data Availability

No datasets were generated or analysed during the current study.

## References

[1] Aires I, Duarte JA, Vitorino R, Moreira-Gonçalves D, Oliveira P, Ferreira R (2024) Restoring skeletal muscle health through exercise in breast cancer patients and after receiving chemotherapy. Int J Mol Sci 25(14):7533. 10.3390/ijms25147533 39062775 10.3390/ijms25147533 PMC11277416

[2] Amaral JF, Mancini M, Novo Júnior JM (2012) Comparison of three hand dynamometers in relation to the accuracy and precision of the measurements. Rev Bras Fisioter 16(3):216–224. 10.1590/s1413-35552012000300007 22801514 10.1590/s1413-35552012000300007

[3] American Society of Hand Therapists (1992) Clinical Assessment Recommendations. American Society of Hand Therapists

[4] Annunziata MA, Muzzatti B, Flaiban C, Gipponi K, Carnaghi C, Tralongo P, Caruso M, Cavina R, Tirelli U (2018) Long-term quality of life profile in oncology: a comparison between cancer survivors and the general population. Supportive Care in Cancer: official journal of the Multinational Association of Supportive Care in Cancer 26(2):651–656. 10.1007/s00520-017-3880-8 28918552 10.1007/s00520-017-3880-8

[5] Bohannon RW, Walsh S (1992) Nature, reliability, and predictive value of muscle performance measures in patients with hemiparesis following stroke. Arch Phys Med Rehabil 73(8):721–725 1642521

[6] Bohannon RW, Peolsson A, Massy-Westropp N, Desrosiers J, Bear-Lehman (2006) Reference values for adult grip strength measured with a Jamar dynamometer: A descriptive meta-analysis. 92(1): 11–15. 10.1016/j.physio.2005.05.003

[7] Bohannon RW, Bear-Lehman J, Desrosiers J, Massy-Westropp N, Mathiowetz V (2007) Average grip strength: a meta-analysis of data obtained with a Jamar dynamometer from individuals 75 years or more of age. J Geriatr Phys Ther 30(1):28–30 19839178

[8] Bohannon RW (2019) Grip strength: an indispensable biomarker for older adults. Clin Interv Aging 14:1681–1691. 10.2147/CIA.S194543 31631989 10.2147/CIA.S194543 PMC6778477

[9] Campos E Silva AC, Bergmann A, Araujo CM, Montenegro AKS, Tenorio ADS, Dantas D (2022) Association of handgrip strength with quality of life in breast cancer survivors: a systematic review and meta-analysis. Asian Pac J Cancer Prev 23(10):3237–3245. 10.31557/APJCP.2022.23.10.3237 36308344 10.31557/APJCP.2022.23.10.3237 PMC9924335

[10] Cantarero-Villanueva I, Fernández-Lao C, Díaz-Rodríguez L, Fernández-de-Las-Peñas C, Ruiz JR, Arroyo-Morales M (2012) The handgrip strength test as a measure of function in breast cancer survivors: relationship to cancer-related symptoms and physical and physiologic parameters. Am J Phys Med Rehabil 91(9):774–782. 10.1097/PHM.0b013e31825f1538 22760108 10.1097/PHM.0b013e31825f1538

[11] Celis-Morales CA, Welsh P, Lyall DM, Steell L, Petermann F, Anderson J et al (2018) Associations of grip strength with cardiovascular, respiratory, and cancer outcomes and all cause mortality: prospective cohort study of half a million UK Biobank participants. BMJ 361:k1651. 10.1136/bmj.k1651 29739772 10.1136/bmj.k1651 PMC5939721

[12] Christensen JF, Jones LW, Andersen JL, Daugaard G, Rorth M, Hojman P (2014) Muscle dysfunction in cancer patients. Annals of Oncology: Official Journal of the European Society for Medical Oncology 25(5):947–958. 10.1093/annonc/mdt551 24401927 10.1093/annonc/mdt551

[13] Courneya KS, Segal RJ, Mackey JR, Gelmon K, Reid RD, Friedenreich CM, Ladha AB, Proulx C, Vallance JK, Lane K, Yasui Y, McKenzie DC (2007) Effects of aerobic and resistance exercise in breast cancer patients receiving adjuvant chemotherapy: a multicenter randomized controlled trial. Journal of Clinical Oncology: Official Journal of the American Society of Clinical Oncology 25(28):4396–4404. 10.1200/JCO.2006.08.2024 17785708 10.1200/JCO.2006.08.2024

[14] Courneya KS, Segal RJ, McKenzie DC, Dong H, Gelmon K, Friedenreich CM, Yasui Y, Reid RD, Crawford JJ, Mackey JR (2014) Effects of exercise during adjuvant chemotherapy on breast cancer outcomes. Med Sci Sports Exerc 46(9):1744–1751. 10.1249/MSS.0000000000000297 24633595 10.1249/MSS.0000000000000297

[15] Crawford J (2019) What are the criteria for response to cachexia treatment? Ann Palliat Med 8(1):43–49. 10.21037/apm.2018.12.08 30685983 10.21037/apm.2018.12.08

[16] Díaz Muñoz G, Callejas Martínez P, Cuesta Malagón V, Calvera Millán SJ (2018) Concordance-Conformity Within Camry and Jamar Hand Dynamometer in Adults. Rev Nutr Clin Metab 1:35–41. 10.35454/rncm.v1n1.075

[17] Dillon HT, Foulkes SJ, Baik AH, Scott JM, Touyz RM, Herrmann J, Haykowsky MJ, La Gerche A, Howden EJ (2024) Cancer therapy and exercise intolerance: the heart is but a part: *JACC: cardiooncology* state-of-the-art review. JACC CardioOncol 6(4):496–513. 10.1016/j.jaccao.2024.04.006 39239327 10.1016/j.jaccao.2024.04.006 PMC11372306

[18] Duarte ACF, Silva BA, Avelino PR, de Menezes KKP (2020) Força de preensão, capacidade funcional e qualidade de vida de indivíduos com câncer. Fisioter Pesqui 27(4):362–369. 10.1590/1809-2950/19039127042020

[19] Güçlüöver A, Kutlu M, Ciğerci AE, Esen HT, Demirkan E, Erdoğdu M (2015) Determination the validity of the new developed Sport Experts® hand grip dynamometer, measuring continuity of force, and comparison with current Takei and Baseline® dynamometers. J Sports Med Phys Fitness 55(11):1318–1321 25289714

[20] Guigni BA, Callahan DM, Tourville TW, Miller MS, Fiske B, Voigt T, Korwin-Mihavics B, Anathy V, Dittus K, Toth MJ (2018) Skeletal muscle atrophy and dysfunction in breast cancer patients: role for chemotherapy-derived oxidant stress. Am J Physiol Cell Physiol 315(5):C744–C756. 10.1152/ajpcell.00002.2018 30207784 10.1152/ajpcell.00002.2018 PMC6293050

[21] Hadzibegovic S, Porthun J, Lena A, Weinländer P, Lück LC, Potthoff SK, Rösnick L, Fröhlich AK, Ramer LV, Sonntag F, Wilkenshoff U, Ahn J, Keller U, Bullinger L, Mahabadi AA, Totzeck M, Rassaf T, von Haehling S, Coats AJS, Anker SD, Roeland EJ, Landmesser U, Anker MS (2023) Hand grip strength in patients with advanced cancer: A prospective study. J Cachex Sarcopenia Muscle 14(4):1682–1694. 10.1002/jcsm.13248 10.1002/jcsm.13248 PMC10401539 37318103

[22] Huang Q, Wu M, Wu X, Zhang Y, Xia Y (2022) Muscle-to-tumor crosstalk: the effect of exercise-induced myokine on cancer progression. Biochimica et Biophysica Acta (BBA) 1877(5):188761. 10.1016/j.bbcan.2022.188761 10.1016/j.bbcan.2022.188761 35850277

[23] Huang L, Liu Y, Lin T, Hou L, Song Q, Ge N, Yue J (2022) Reliability and validity of two hand dynamometers when used by community-dwelling adults aged over 50 years. BMC Geriatr 22(1):580. 10.1186/s12877-022-03270-6 35840905 10.1186/s12877-022-03270-6 PMC9284760

[24] Jiménez-Sánchez A, Pereira-Cunill JL, Limón-Mirón ML, López-Ladrón A, Salvador-Bofill FJ, García-Luna PP (2024) A cross-sectional validation study of Camry EH101 versus JAMAR Plus hand-held dynamometers in colorectal cancer patients and their correlations with bioelectrical impedance and nutritional status. Nutrients 16(12):1824. 10.3390/nu16121824 38931179 10.3390/nu16121824 PMC11206484

[25] Jochem C, Leitzmann M, Volaklis K, Aune D, Strasser B (2019) Association between muscular strength and mortality in clinical populations: a systematic review and meta-analysis. J Am Med Dir Assoc 20(10):1213–1223. 10.1016/j.jamda.2019.05.015 10.1016/j.jamda.2019.05.015 31331825

[26] Klassen O, Schmidt ME, Ulrich CM, Schneeweiss A, Potthoff K, Steindorf K, Wiskemann J (2017) Muscle strength in breast cancer patients receiving different treatment regimes. J Cachexia Sarcopenia Muscle 8(2):305–316. 10.1002/jcsm.12165 27896952 10.1002/jcsm.12165 PMC5377413

[27] Konieczny M, Cipora E, Roczniak W, Babuśka-Roczniak M, Wojtaszek M (2020) Impact of time to initiation of treatment on the quality of life of women with breast cancer. Int J Environ Res Public Health 17(22):8325. 10.3390/ijerph17228325 33187071 10.3390/ijerph17228325 PMC7696805

[28] Kottner J, Audige L, Brorson S et al (2011) Guidelines for reporting reliability and agreement studies (GRRAS) were proposed. Int J Nurs Stud 48:661–671. 10.1016/j.jclinepi.2010.03.002 21514934 10.1016/j.ijnurstu.2011.01.016

[29] Lakoski SG, Eves ND, Douglas PS, Jones LW (2012) Exercise rehabilitation in patients with cancer. Nat Rev Clin Oncol 9(5):288–296. 10.1038/nrclinonc.2012.27 22392097 10.1038/nrclinonc.2012.27 PMC3640332

[30] Magni N, Olds M, McLaine S (2023) Reliability and validity of the K-force grip dynamometer in healthy subjects: do we need to assess it three times? Hand Ther 28(1):33–39. 10.1177/17589983231152958 37904810 10.1177/17589983231152958 PMC10584072

[31] Mathiowetz V, Weber K, Volland G, Kashman N (1984) Reliability and validity of grip and pinch strength evaluations. J Hand Surg 9(2):222–226. 10.1016/s0363-5023(84)80146-x 10.1016/s0363-5023(84)80146-x 6715829

[32] Matsui R, Inaki N, Tsuji T (2021) The impact of the preoperative hand grip strength on the long-term outcomes after gastrectomy for advanced gastric cancer. Surg Today 51(7):1179–1187. 10.1007/s00595-021-02256-y 33713199 10.1007/s00595-021-02256-y

[33] Merry K, Napier C, Chung V, Hannigan BC, MacPherson M, Menon C, Scott A (2021) The validity and reliability of two commercially available load sensors for clinical strength assessment. Sensors 21(24):8399. 10.3390/s21248399 34960492 10.3390/s21248399 PMC8703969

[34] Mitchell WK, Williams J, Atherton P, Larvin M, Lund J, Narici M (2012) Sarcopenia, dynapenia, and the impact of advancing age on human skeletal muscle size and strength; a quantitative review. Front Physiol 3:260. 10.3389/fphys.2012.00260 22934016 10.3389/fphys.2012.00260 PMC3429036

[35] Mokkink LB, Terwee CB, Patrick DL, Alonso J, Stratford PW, Knol DL, Bouter LM, de Vet HC (2010) The COSMIN checklist for assessing the methodological quality of studies on measurement properties of health status measurement instruments: an international Delphi study. Qual Life Res 19(4):539–549. 10.1007/s11136-010-9606-8 20169472 10.1007/s11136-010-9606-8 PMC2852520

[36] Mokkink LB, Prinsen CA, Bouter LM, Vet HC, Terwee CB (2016) The consensus-based standards for the selection of health measurement instruments (COSMIN) and how to select an outcome measurement instrument. Braz J Phys Ther 20(2):105–113. 10.1590/bjpt-rbf.2014.0143 26786084 10.1590/bjpt-rbf.2014.0143 PMC4900032

[37] Nikodelis T, Savvoulidis S, Athanasakis P, Chalitsios C, Loizidis T (2021) Comparative study of validity and reliability of two handgrip dynamometers: K-force grip and Jamar. Biomechanics 1(1):73–82. 10.3390/biomechanics1010006

[38] Paek J, Choi YJ (2019) Association between hand grip strength and impaired health-related quality of life in Korean cancer survivors: a cross-sectional study. BMJ Open 9(9):e030938. 10.1136/bmjopen-2019-030938 31501128 10.1136/bmjopen-2019-030938 PMC6738715

[39] Pereira AAC, Zaia RD, Souza GHG, Luizeti BO, Andreola R, Junior AOV, Ferrari A (2021) The correlation between hand grip strength and nutritional variables in ambulatory cancer patients. Nutr Cancer 73(2):221–229. 10.1080/01635581.2020.1750662 32286094 10.1080/01635581.2020.1750662

[40] Pino-Ortega J, Carvajal-Espinoza R, Becerra-Patiño BA (2024) Evaluation of hand muscle strength using manual dynamometry: a reliability and validity study of the Activ5 instrument. Appl Sci 14(19):8775. 10.3390/app14198775

[41] Rijk JM, Roos PR, Deckx L, van den Akker M, Buntinx F (2016) Prognostic value of handgrip strength in people aged 60 years and older: a systematic review and meta-analysis. Geriatr Gerontol Int 16(1):5–20. 10.1111/ggi.12508 26016893 10.1111/ggi.12508

[42] Roberto M, Barchiesi G, Resuli B, Verrico M, Speranza I, Cristofani L, Pediconi F, Tomao F, Botticelli A, Santini D (2024) Sarcopenia in breast cancer patients: a systematic review and meta-analysis. Cancers (Basel) 16(3):596. 10.3390/cancers16030596 38339347 10.3390/cancers16030596 PMC10854936

[43] Savas S, Kilavuz A, Kayhan Koçak FÖ, Cavdar S (2023) Comparison of grip strength measurements by widely used three dynamometers in outpatients aged 60 years and over. J Clin Med 12(13):4260. 10.3390/jcm12134260 37445293 10.3390/jcm12134260 PMC10342845

[44] Schmid D, Leitzmann MF (2014) Association between physical activity and mortality among breast cancer and colorectal cancer survivors: a systematic review and meta-analysis. Official journal of the european society for medical oncology. Ann Oncol 25(7):1293–1311. 10.1093/annonc/mdu012 10.1093/annonc/mdu012 24644304

[45] Schmidt ME, Wiskemann J, Steindorf K (2018) Quality of life, problems, and needs of disease-free breast cancer survivors 5 years after diagnosis. Qual Life Res 27(8):2077–2086. 10.1007/s11136-018-1866-8 29740782 10.1007/s11136-018-1866-8

[46] Schneider CM, Hsieh CC, Sprod LK, Carter SD, Hayward R (2007) Cancer treatment-induced alterations in muscular fitness and quality of life: the role of exercise training. Ann Oncol 18(12):1957–1962. 10.1093/annonc/mdm364 17804476 10.1093/annonc/mdm364

[47] Tuttle CSL, Thang LAN, Maier AB (2020) Markers of inflammation and their association with muscle strength and mass: a systematic review and meta-analysis. Ageing Res Rev 64:101185. 10.1016/j.arr.2020.101185 32992047 10.1016/j.arr.2020.101185

[48] Ulrike Sonja, Trampisch Julia, Franke Nina, Jedamzik Timo, Hinrichs Petra, Platen (2012) Optimal Jamar Dynamometer Handle Position to Assess Maximal Isometric Hand Grip Strength in Epidemiological Studies The Journal of Hand Surgery 37(11) 2368-2373 10.1016/j.jhsa.2012.08.014 10.1016/j.jhsa.2012.08.014 23101534

[49] Villaseñor A, Ballard-Barbash R, Baumgartner K, Baumgartner R, Bernstein L, McTiernan A, Neuhouser ML (2012) Prevalence and prognostic effect of sarcopenia in breast cancer survivors: the HEAL study. J Cancer Surviv 6(4):398–406. 10.1007/s11764-012-0234-x 23054848 10.1007/s11764-012-0234-x PMC3747827

[50] World Medical Association (2014) Declaration of Helsinki: ethical principles for medical research involving human subjects. J Am Coll Dent 81:14–18 25951678

